# ‘A good ending but not the end’: Exploring family preparations surrounding a relative’s death and the Afterlife – A qualitative study

**DOI:** 10.1177/02692163241280016

**Published:** 2024-09-27

**Authors:** Hui-Ju Liang, Qian Xiong, Peng-Chan Lin, Jui-Hung Tsai, Nancy Preston

**Affiliations:** 1 Division of Health Research, Faculty of Health and Medicine, Lancaster University, Health Innovation One, Lancaster, UK; 2 International Observatory on End of Life Care, Division of Health Research, Lancaster University, Lancaster, UK; 3 Centre for Ageing Research, Division of Health Research, Faculty of Health and Medicine, Lancaster University, Lancaster, UK; 4 Department of Oncology, National Cheng Kung University Hospital, College of Medicine, National Cheng Kung University, Tainan, Taiwan; 5 Centre for Hospice Palliative Shared Care, National Cheng Kung University Hospital, College of Medicine, National Cheng Kung University, Tainan, Taiwan

**Keywords:** Palliative care, death, family, religion, culture, funeral ceremony, qualitative research

## Abstract

**Background:**

Adequate death preparation positively influences families’ experience before death and during bereavement. However, how to prepare families in non-Western cultures has received scant attention.

**Aim:**

To explore family caregivers’ experiences in preparing for a relative’s death in specialist palliative care in Taiwan.

**Design:**

A qualitative study employing reflexive thematic analysis of data collected from semi-structured interviews was conducted.

**Setting/participants:**

Twenty-two family caregivers from seven hospitals participated.

**Results:**

The overarching theme was ‘getting everything right to have no regrets between the dead and the living’. We developed two themes to explain preparations for the time surrounding and after the death, including the deceased’ afterlife: (1) ‘having a good ending but not the end of the relationship’, which addresses preparations for the death itself, the funeral, the afterlife and maintaining connections and (2) ‘using religious beliefs and cultural norms to guide preparation’, which explores perceptions of a good death, including refrain from strong emotions before and after the death.

**Conclusion:**

Funeral arrangements, enhancing the deceased’s afterlife and maintaining connections to the deceased are crucial for families’ experiences which can be impacted by actions they take as they prepare for the death. A culturally appropriate death is beneficial for the dying relative which includes preparing to not show strong emotions during and after the death. These insights inform the importance of the cultural context in death preparation in Taiwan and provide perspectives for palliative care beyond Western culture, potentially benefiting Chinese populations, predominantly East Asian and Buddhist societies.

**What is already known about the topic?**

- Preparation for a relative’s death is crucial in palliative and end-of-life care, greatly influencing the experience of family members both before the death and during subsequent bereavement.
- Preparedness, including cognitive, behavioural and emotional readiness, is a multifaceted concept requiring accomplishment through medical, psychosocial, spiritual and practical tasks.
- Research on death preparation, predominantly conducted in Western countries, may limit its universal applicability, hindering the provision of culturally appropriate palliative and end-of-life care.

**What this paper adds?**

- Preparing for the time after death is crucial in Taiwanese families, including appropriate conduct at the moment of death, organising a meaningful funeral, enhancing the deceased’s afterlife and preparing for ongoing relationships.
- Taiwanese families’ preparation for death emphasises achieving a culturally appropriate death for the dying relative, with a focus on a dignified appearance (clean and well-dressed body, eyes closed, mouth shut) at the moment of death and ensuring a better afterlife, aiming to prevent regrets for both the deceased and the living.
- Preparing to refrain from strong emotions around the dying relative, both during and after their death, is important and culturally appropriate and believed to benefit their well-being.

**Implications for practice, theory or policy**

- Understanding and respecting families’ religious beliefs (e.g. regarding the afterlife) and conforming to cultural norms are essential when assisting them in preparing for a relative’s death.
- Our study contributes to a deeper understanding of what constitutes a good death from family perspectives within Taiwan’s cultural context, which may apply to other Chinese societies.
- Further research could explore culturally appropriate emotional support strategies for Taiwanese individuals and develop effective approaches to establishing continuing relationships before a relative’s death that positively impact bereavement adjustments, thereby informing proactive bereavement support.

## Introduction

Preparedness for a relative’s death involves cognitive, behavioural and emotional readiness^1–3^ and requires managing medical, psychosocial, spiritual and practical tasks.^2–4^ Assisting families in this is crucial in palliative and end-of-life care,^5,6^ with evidence showing positive impacts on families, including emotional support, acceptance of death^7^ and better bereavement outcomes such as lower levels of grief^8^ and reduced complicated grief.^1,8–10^ Despite its importance, there is a need to improve this aspect of care as studies indicate that family caregivers often lack essential information about the dying process and are unsure about the appropriate actions to take at the time of death,^11^ with approximately 60% (*N* = 393) feeling inadequately prepared.^12^

Research on families’ death preparation remains understudied, with most focussing on Western countries like the United States,^2,7,13,14^ Canada^15^ and Australia.^16^ Culture influences how people approach death, respond to related issues and define a good or bad death.^17^ Differences in culture suggests that findings from Western studies may not universally apply, potentially hindering culturally appropriate palliative and end-of-life care for other groups. Therefore, research specific to non-Western cultures, like East Asian contexts, is essential to address this gap.

Although some studies in East Asian cultures have explored preparedness for death, they often do not fully capture the comprehensive actions taken by families. For example, research in Taiwan has examined how family caregivers’ preparedness relates to variables like depressive symptoms,^18–20^ quality of life,^18^ prolonged grief symptoms,^20^ and subjective caregiving burden.^19^ In Japan, insufficient preparedness has been linked to factors like unawareness of patients’ medical conditions.^21^ Given the influence of Confucianism and Collectivism in East Asian cultures, which emphasise familial support, filial piety (children showing respect, obedience and care for parents), mutual dependence,^22^ and concern for others,^23^ means culturally contextualised studies are needed to better understand death preparation in the region.

Taiwan ranks the third among the 81 countries in the 2021 Quality of Death and Dying study,^24^ offering a well-developed palliative care system to evaluate death preparation in non-Western cultures. With constitutionally protected freedom of religion, Taiwan’s diverse religious landscape, including Buddhism, Taoism, Christianity and Taiwanese folk religion (a blend of Buddhism, Taoism and Confucianism),^25^ offers insights into the role of religion in death preparation. This study explores how cultural context influences family caregivers’ experiences of preparing for a relative’s death in specialist palliative care in Taiwan.

## Methods

### Design

The study employed Braun and Clarke’s reflexive thematic analysis with a Big Q approach grounded in critical realism,^26^ highlighting the acknowledgement that reality exists but is subject to contextual influences.^27^ This method was chosen for its theoretical flexibility and alignment with the study’s critical realism philosophy,^26^ aiding in understating participants’ experiences within a broader context by developing meaningful patterns across the dataset.^26,28^ The inductive nature informs both data collection and analysis. Reporting of the study followed the Reflexive Thematic Analysis Reporting Guidelines.^26,29–31^

### Setting

In Taiwan, government-funded palliative care services are integrated into the healthcare system to support patients with a cancer or non-cancer diagnosis. Specialist palliative care is provided by multidisciplinary teams with advanced training, operating across hospice inpatient units, consultation teams and home care services,^32^ which is the context of this study.

### Participants

Eligible participants were bereaved family members of patients who received specialist palliative care services before their death. They were aged 20 years or older, fluent in Mandarin or Taiwanese, and served as unpaid caregivers. The bereaved timeframe ranged from 6 to 18 months before recruitment, following previous studies on preparedness.^13,14^ Six months post-death allowed for the grieving process,^33^ while the 18-month limit facilitated recall of experiences. Those bereaved by a child’s death (under 20 years old) were excluded due to their unique experiences, as indicated by previous research.^34^

### Sampling and recruitment

Purposive and snowballing sampling methods were employed to recruit participants. Specialist palliative care teams, who maintain records of deceased patients to support bereaved families, were contacted to assist recruitment. Each potential participant received an information sheet and consent form (Supplemental Appendix 1). Interested individuals contacted the research team or had their information forwarded if preferred. The consent process included clarifications and additional details as requested. Written informed consent was obtained before the interview in-person or electronically.

### Data collection

Semi-structured interviews were conducted from October 2022 to March 2023 using a literature-informed topic guide,^2,7,13–15^ covering awareness of impending death, challenges, facilitators, tasks and emotions related to death preparation (Supplemental Appendix 2). Minor adjustments were made following a pilot interview in September 2022, including adding a question about actions that enhance preparedness and those to avoid.

H-JL conducted individual interviews in person or virtually, based on participants’ preferences and COVID-19 policies. In-person interviews occurred at participants’ homes or in private public spaces like cafes. An interview distress protocol^35^ (Supplemental Appendix 3) was developed. H-JL maintained a reflexive journal, reflecting on assumptions, emotions and thoughts.^26^

Following the study’s philosophical assumptions, when recruitment ended was guided by information power, ensuring data sufficiency for addressing the research questions. The assessment was ongoing through data collection and analysis.^26,36^

### Ethical approval

Approval was obtained from the Hospital (A-ER-111-193) and University (FHM-2022-0972-ExRev-1) Research Ethics Committee.

### Data analysis

The six-phase recursive process of reflexive thematic analysis was employed.^26^ H-JL transcribed interview audio recordings in Chinese, managing with NVivo software. Coding was conducted in Chinese for linguistic convenience and data proximity but with some initial translation for discussion with the wider team as NP did not speak Chinese.^37^ H-JL conducted two rounds of inductive coding, generating distinct code labels without predetermined frames. The list of code labels was translated into English, keeping certain original Chinese words by the end of coding. H-JL developed initial themes by clustering related codes, refining them through review and thematic maps (Supplemental Appendix 4). Continuous discussions with NP and QX aimed to enhance understanding, interpretation and researcher reflexivity.^26^

## Findings

### Participants characteristics

Twenty-two participants (8 men and 14 women) were interviewed from seven hospitals (Table 1), comprising four medical centres, two regional hospitals and one district hospital across Southern (*n* = 3), Central (*n* = 2), Northern (*n* = 1) and Eastern (*n* = 1) Taiwan. Participants’ average age was 55.3 years (range = 23–78 years). Most were women, working full time, adult children of deceased patients and practicing Taiwanese folk religion. On average, they were bereaved for 10.7 months (range = 6–17 months). A total of 21 deceased patients averaged 70.9 years old (range = 44–93 years), mostly women, diagnosed mainly with cancer, with about half dying at home. Interviews lasted between 70 and 186 min, averaging 115 min.

**Table 1.** Descriptive characteristics of family members and deceased patients.

Family member participant characteristics	*N* = 22
Age (years)	
20–40	2
41–65	15
66+	5
Gender	
Man	8
Woman	14
Employment status	
Full time	12
Retired	8
Unemployed	2
Religious beliefs	
Taiwanese folk religion	8
Buddhism	3
Taoism/Daoism or Yiguandao	3
Christianity/Catholic	6
No affiliation	2
Relationship with deceased patients	
Adult children	14
Spouse	5
Sibling	2
Parents of adult child	1
Bereaved time before recruitment (months)	
6–12	18
13–18	4
Deceased patient characteristics	*N* = 21
Age at death (years)	
41–65	7
66–80	9
81+	5
Gender	
Man	4
Woman	17
Primary medical diagnosis	
Cancer	14
Non-caner	7
Specialist palliative care received before death	
Inpatient, consultation and home care	7
Inpatient and consultation care	4
Inpatient and home care	3
Inpatient care	2
Home care	5
Death of place	
Home	10
Hospital	11

### Themes

**Figure 1.**
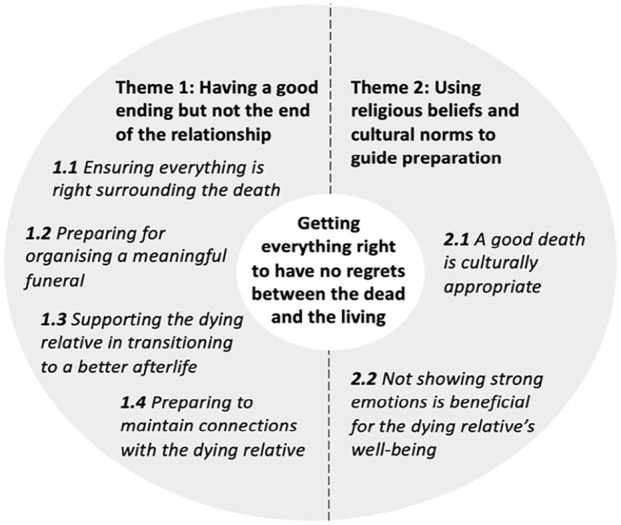
Families’ preparations for the time surrounding and after a relative’s death.

The overarching theme was ‘getting everything right to have no regrets between the dead and the living’, a primary goal in Taiwanese families’ death preparation. Families strived to ensure a culturally appropriate death for their dying relative, aiming to avoid regrets. We developed two themes to explain families’ preparations for the time surrounding and after the death, including the deceased’ afterlife: ‘having a good ending but not the end of the relationship’ and ‘using religious beliefs and cultural norms to guide preparation’ (Figure 1). These preparations and beliefs about the afterlife hold specific cultural importance in Taiwanese families’ approach to a relative’s death, which is discussed below.

#### Theme 1: Having a good ending but not the end of the relationship

Taiwanese families’ death preparation extended beyond the moment of death, including actions from the dying process through to the funeral. These preparations aimed to ensure a good ending, involving appropriate conduct at the moment of death, arranging a meaningful funeral and enhancing a better afterlife. Emphasis was also placed on preparing to maintain connections with the deceased.

##### Ensuring everything is right surrounding the death

Families ensured appropriate conduct at the moment of death by being physically present and ensuring the dying relative had a clean, neatly dressed body to uphold their dignity. Preparing to be physically present at the death involved learning to recognise dying signs, planning arrival times, deciding on attendees and considering alternative methods if unable to be present. This presence fostered closeness, allowing final interactions and farewells and ensuring the relative did not die alone, with immediate and extended family typically present:

*In total, more than 30 relatives, including my aunts and sisters, came to see my mother for the final time, to say the last goodbye. (Son, FC16)*

Video calls often served as an alternative for this final interaction, especially during the pandemic when physical presence was hindered by quarantine policies. However, despite offering comfort, this method cannot fully replace physical presence, which might be more helpful in later bereavement.

Preparing a clean, neatly dressed body and offering comforting words were crucial for ensuring the dying relative could ‘*depart with dignity’ (Daughter, FC2)*. This involved preparing clothing and necessary items, understanding body handling procedures at death, including managing tubes like ‘*urinary catheters’ (Husband, FC15)* for home deaths, and learning how to clean the relative’s body and dress them, often with coordination from nurses or funeral staff:

*At the last moment, when we were changing his clothes, my son helped shave his beard and tidy up his hair. The hospice nurse assisted us with this. (Wife, FC1)*

##### Preparing for organising a meaningful funeral

Getting the funeral ‘right’ was essential and required advance planning. In Taiwanese culture, it is a crucial life event symbolising life’s conclusion. A well-conducted funeral offered the deceased a dignified ending and insights into their life. For instance, a large funeral could symbolise societal contributions and convey love, echoing the Taiwanese saying, ‘*As the coffin is closed, the fate is sealed*’ *(蓋棺論定, Gài guān lùn ding) (Daughter, FC18)*, which reflects beliefs that judgments on one’s life are made after death. Families also expressed gratitude and love through funeral activities, contributing to overall preparation. A common and unique practice involved collaborating with funeral staff to prepare the deceased’s body before the ceremony:

*The funeral staff asked me if I would like to come and watch as they gave my mum a spa treatment. I agreed. During this process, he guided me to give my mum a massage, massaging her hands and feet. He also guided me to kiss her and say goodbye. In that process, I could still express to my mum; I love you [chopped up and teary-eyed], and say goodbye while talking to her. (Daughter, FC7)*

Funeral preparations were intricate, involving decisions on whether, who, what and how to discuss arrangements with the dying relative. Some families chose open engagement, while others opted to shield the dying relative. Selecting the ‘right’ individuals, like those perceived as ‘*better at communication*’ *(Daughter, FC4)* and possessing ‘*more rational and positive thinking*’ *(Sister, FC5)*, was crucial. Discussions varied from discussing key principles to planning detailed tasks such as organising elaborate *‘burial clothes’ (Daughter, FC2)* and *‘posthumous photos’ (Wife, FC1)*, choosing a *‘funeral venue’ (Daughter, FC4)* and *‘resting place’ (Husband, FC15)*, arranging *‘religious rituals’ (Daughter, FC3)*, preparing offerings like *‘flowers’ (Daughter, FC2)* and *‘white envelopes’ (白包, Bái bāo, a Taiwanese tradition involving monetary gifts) (Son, FC21)*. An invitation list was compiled based on the preferences of the family and their dying relative. Using appropriate language and alternative expressions to address sensitive topics was vital during these discussions. For instance, asking questions like ‘*Where would you like to live afterwards?’ (Daughter, FC18)* instead of directly discussing burial places helped navigate the conversation thoughtfully.

##### Supporting the dying relative in transitioning to a better afterlife

Preparing for a better afterlife was integral to a good ending, influenced by religious beliefs and cultural customs. This included guiding the dying relative towards their destination after death, facilitating a smooth transition and alleviating suffering in the afterlife.

Preparing the dying relative for their destination after death involved discussing shared religious beliefs about the afterlife. Religious expressions like encouraging them to ‘*follow the Bodhisattva’ (菩薩, Púsà, referring to a being committed to the path of a Buddhahood) (Daughter, FC6)* or assuring them with statements such as *‘God loves you, you do not have to be afraid and follow the light’ (Daughter, FC9)* were commonly used in their final moments.

Preparing for a smooth transition from life to death involved contacting religious practitioners like ‘*the head monk of the temples’ (Daughter, FC17)* or *‘a minister in Christianity’ (Daughter, FC9)* in advance. For Buddhists and followers of Taiwanese folk religion, who believed *‘the soul experienced great pain during separation from the body’ (Son, FC16)*, the *‘support-chanting’ (助念, Zhù niàn) (Son, FC11)* ritual was arranged. This ritual, conducted from dying until hours after death, involved ***‘*** *chanting Buddhist names’ (Son, FC16)* and *‘avoiding moving the body’ (Son, FC11)*. Preparations included *‘organising contact details for chanting groups’ (Son, FC21)*, preparing the *‘Rebirth Blanket’ (a Buddhist item for alleviating suffering, bringing blessings and guiding the deceased to rebirth in the Pure Land) (Husband, FC20)* and discussing the location and duration of the chanting session.

Ritual preparations from the deathbed to the funeral aimed to enhance the deceased’s afterlife, ensuring essentials like ‘*money in the afterworld’ (Wife, FC1)*. Rooted in Taiwanese folk religion, common practices included burning *‘paper gold ingots, paper lotus flowers’ (Wife, FC1)*, and *‘symbolic daily necessities like paper watches’ (Daughter, FC2)*. Influenced by Confucianism, there was a tradition of preparing the deceased’s favourite food as sacrificial offerings:

*During the funeral period, we needed to worship him three times a day, ensuring that he could have a meal with each of them. (Daughter, FC4)*

##### Preparing to maintain connections with the dying relative

Ongoing care for the afterlife reflected families’ belief in a lasting connection. Before death, it was important to prepare for a continuing relationship with the deceased. Non-religious methods included ‘*leaving behind some memories*’ *(Daughter, FC9)* and preparing physical items like *‘photos’ (Daughter, FC17), ‘videos’ (Wife, FC1), ‘gifts’ (Daughter, FC9)* and creative works such as *‘handprint models’ (Daughter, FC12)*. Viewing mementoes provided a sense of the deceased’s enduring presence; as one participant described, ‘I*t made me feel like our mother was still accompanying us’ (Daughter, FC9)*. However, allocating sufficient time and emotional energy for this viewing mementoes after death was essential:

*I actually don’t dare to look at my deceased father’s photos and videos. I recorded a lot of them, but I dared not watch them after he left [referring to dying]. This week, I happened to reset my mobile phone, so basically, everything I recorded was gone. I backed them up on my laptop, but they were completely detached from my mobile phone. I’m not ready to watch them. (Son, FC11)*

Anticipating a future reunion brought comfort, providing a sense of being *‘a bit better and relaxed’ (Daughter, FC12)* during the death preparation. Rooted in religious beliefs, Buddhists anticipated reunion in the *‘Western Pure Land’ (Daughter, FC17)*, while Christians in *‘Heaven’ (Sister, FC8)*. Discussing the future reunion with the dying relative before death was helpful in preparing for a continuing relationship afterwards:

*I knew my elder sister told her, you’ve gone, and we will follow. In the next life, we’ll meet again. Go in peace, and we’ll be sisters again in the next life [choking up and shedding tears]. (Sister, FC5)*

Religious beliefs and cultural norms influenced families’ preparations and actions from the dying process to the funeral, focussing on ensuring a better afterlife and maintaining connections with the deceased, as discussed above. The following theme further explores this influence.

#### Theme 2: Using religious beliefs and cultural norms to guide preparation

Religious beliefs and cultural norms guided Taiwanese families in preparing for a relative’s death. This theme explores how these beliefs shaped families’ perceptions of a good death and influenced their emotional expressions before and after the death.

##### A good death (‘好走’, Hǎo zǒu) is culturally appropriate

Achieving a culturally appropriate death (‘好走’, Hǎo zǒu) was crucial in families’ death preparations, ensuring no regrets between the deceased and the living. Families emphasised following religious and cultural beliefs to allow the dying relative to die naturally, such as *‘allowing nature to take its course’ (順其自然, shùn qí zì rán) (Wife, FC1), ‘obeying destiny’ (聽天命, tìng tiān mìng) (Sister, FC5)* and accepting that *‘the lifespan of human beings has been predestined’ (Son, FC22)* by the Creator/God/gods. These beliefs helped families accept imminent death and enhanced their emotional readiness:

*It’s just that the determination of time is in God’s arrangement. . . . So, what I meant was, when my wife left [referring to dying], it was also her time, and she departed. It’s the same for everyone. This was also to prepare my sons psychologically. (Husband, FC19)*

Some specific indicators at the moment of death shaped a culturally appropriate death, reflecting careful preparations discussed in Theme 1. Physical presence of relatives at the moment of death, influenced by the cultural tradition of ‘Suí shì zài cè’ (隨侍在側, signifying descendants being at the bedside), was crucial, especially for senior relatives:

*I felt that my mother had a peaceful departure because all her children were by her side at her dying, which I believed was very comforting. People often talk about having a good death, where your descendants surround you during your dying, and you can peacefully rest. For me, my mother’s dying was like this – a good and peaceful ending. (Daughter, FC7)*

Emphasis was also placed on a dignified appearance at the moment of death, which included a clean and well-dressed body, a peaceful expression resembling sleep with *‘no pain’ (Son, FC21), ‘no discomfort’ (Wife, FC14)* and *‘eyes closed and mouth shut’ (Daughter, FC18)*. These aspects echoed the cultural belief of ‘sǐ yě míng mù (死也瞑目), symbolising a peaceful death free from worries and attachments, articulated as *‘no attachments left’ (Son, FC21)* and *‘wasn’t afraid’ (Daughter, FC17)*.

A better, suffering-free afterlife was integral to a culturally appropriate death. Families believed the deceased transitioned to this favourable afterlife, often recognised it when the deceased’s body remained *‘still soft’ (Daughter, FC7)*, influenced by their religious beliefs:

*My mother appeared lively, not like a dead person; her hands could be lifted, and her body was soft as cotton after being taken out of the freezer. I felt she had departed from the sea of suffering [苦海, Kǔhǎi, referring to the life of a human being is filled with various hardships and pains]. She ascended to the Boundless Pure Land [referring to an after-world advocated by Yiguandao – a religion which combines the five teachings, including Confucianism, Taoism, Buddhism, Christianity and Islam], commonly known as heaven. Isn’t that wonderful? She is happier there, free from troubles and worries. (Son, FC22)*

Influenced by the cultural belief of *‘leaves falling back to the roots’ (落葉歸根, luò yè guī gēn) (Son, FC16)*, dying at home was considered a good death. When this was not possible, families performed a ritual of *‘covering the deceased with an oxygen mask and waiting to remove it until returning home’ (Son, FC16)*. This required coordination with funeral staff and advance preparation. However, some perceived home deaths unfavourably, fearing a negative impact on the well-being of surviving family members:

*My mother deeply believed that people should not pass away at home. She thought that leaving in such a way would be unfavourable for the people at home, for the family. She believed it would not be good for the younger generations and would be detrimental to the household. (Daughter, FC4)*

##### Not showing strong emotions is beneficial for the dying relative’s well-being

Religious beliefs and cultural norms guided families to control emotional expression for their dying relative’s well-being. Avoiding strong emotions like crying loudly around the dying relative during the dying process and funeral was believed to facilitate a smoother transition and enhance the afterlife, contributing to a good death. However, expressing sorrow through weeping or tears was acceptable. Rooted in Buddhist beliefs, loud crying might hinder the transition:

*I only hoped that she could peacefully and smoothly reach Amitabha Buddha. My belief was so clear. So I didn’t cry. I couldn’t cry. If I cried, what if she fell down? It’s because my religion tells me not to cry. If I cried, it would hinder her. (Daughter, FC17)*

Guided by the collectivist value of concern for others, families practiced emotional restraint to prioritise the dying relative’s needs, aiming to protect them from emotional burdens like feeling *‘uneasy and uncomfortable’ (Husband, FC20)* and to ensure a dignified funeral and a better afterlife:

*Was my mother happy to see me sad? Consider whether my mum would have wanted to see me sad and discouraged because of her leaving [referring to dying]. Was it helpful for my mum if I were sad for her? It might make her feel distressed if she sees me sad from above [referring to an after-world the deceased has reached]. She would worry about me instead. (Son, FC22)*

Religious beliefs and cultural norms shaped views of a good death, promoting emotional restraint before and after death for the well-being of the dying relative.

## Discussion

### Main findings of the study

Preparing for post-death arrangements was crucial for families in Taiwan, aligning with religious beliefs and cultural norms. This involved ensuring appropriate conduct at the moment of death, organising a meaningful funeral, enhancing the deceased’s afterlife and maintaining connections with the deceased. These components comprised a culturally appropriate death. Preparing to not show strong emotions around the dying relative during and after death was necessary to benefit their well-being.

### What this study adds

Our study’s main findings differ from the existing literature, which primarily focusses on death preparation in Western cultures.^2,7,13–16^ In Taiwan, preparing for the funeral was key.^2,16^ Rooted in the Confucian cultural principle of ‘carefully handling the conclusion of life’ (慎終, shèn zhōng), the funeral held diverse meanings, providing closure and insights to the deceased, with a particular emphasis on conducting appropriate funeral ceremonies, especially for parents. Our study underscores the complexity of funeral preparations, involving decisions about the dying relative’s involvement, selecting ‘right’ family members for discussions, determining preparation content and using appropriate language. It challenges the common avoidance of death conversations in Chinese culture, often deemed taboo due to its association with bad luck.^38^ Thus, acknowledging and respecting family culture when addressing death-related topics becomes imperative, demanding a sensitive approach.^2,4^ Recognising funeral preparations is crucial in Taiwan, providing insights into culturally appropriate palliative and end-of-life care, potentially applicable to Confucian-influenced societies like Chinese populations and East Asian countries.^39^

The study underscores the importance of achieving a culturally appropriate death in Taiwanese families’ preparations, focussing on natural death^40^ and freedom from pain,^41–45^ which is consistent with previous evidence. Significant Cultural aspects included ensuring a dignified appearance at death (clean and well-dressed body, eyes closed, mouth shut) and enhancing the afterlife, reflecting Taiwanese religious beliefs and traditional Chinese values of posthumous care.^38^ Despite Taiwan developing a Good Death scale based on Western literature and expert opinions in palliative care, it overlooks these religious beliefs and familial concerns about the afterlife.^46^ Additionally, previous studies have debated whether dying at home effectively indicates a good death.^42,44,47^ with our findings revealing variability based on patient needs and family beliefs. This research sheds light on what constitutes a good death from Taiwanese family perspectives, with implications for similar practices in other Chinese societies. Considering the place of death is crucial in supporting families during death preparation. Further research could investigate whether home deaths reliably reflect good deaths and evaluate the quality of palliative and end-of-life care in Taiwanese populations.^48^

Our study highlights how religious beliefs and cultural norms shaped Taiwanese families’ approach to death preparation, though distinguishing their individual influences was challenging. For instance, refraining from loud crying before and after death was seen as both religiously and culturally appropriate. Some participants engaged in rituals like support-chanting more due to cultural norms than personal religious beliefs.^49^ Moreover, many Taiwanese people follow multiple religions, such as identifying as Buddhists or Taoists while also incorporating beliefs of Taiwanese folk religion.^25^ These intertwined practices and beliefs complicate clear distinctions. Despite this complexity, it remains crucial to understand families’ religious beliefs and respect their cultural norms when assisting with death preparation.

The need to prepare for an ongoing relationship after death is linked to the continuing bonds theory within Western-oriented bereavement theory.^50,51^ The theory, inspired by Japanese ancestor rituals, stresses maintaining connections with the deceased is normal and prevalent.^52^ Despite literature often overlooking this aspect before death,^53,54^ our study reveals how such bonds were formed through actions like creating positive memories^13^ and preparing mementoes with the deceased.^55^ Anticipating and discussing a future reunion with the dying relative, influenced by participants’ beliefs about the afterlife, aided family preparedness, highlighting the importance of aligning strategies with religious beliefs and cultural norms.^53^ Research highlights the importance of maintaining connections in Taiwanese family members’ bereavement experiences,^56^ but the impact of these connections on bereavement adjustment remains debated.^56–61^ Consequently, further research could investigate strategies for maintaining a continuing relationship before death that positively impacts bereavement outcomes, enhancing death preparation and proactive bereavement support. Understanding families’ religious beliefs, particularly about the afterlife, is essential for maintaining connections through religion and improving their preparedness.

Our study highlights the importance of preparing to refrain from strong emotions around the dying relative during and after the death, mainly influenced by Buddhism and the cultural value of collectivism.^23^ A review confirms that hiding emotions is culturally appropriate and linked to religious beliefs during bereavement in Taiwan.^56^ However, expressing emotions is generally seen as adaptive in Western literature during grieving.^60,62,63^ Further research could explore culturally appropriate emotional support strategies for Taiwanese people, Chinese populations and individuals following Buddhism.

### Reflections, strengths and limitations of the study

The main researcher (H-JL), a senior palliative care nurse from Taiwan, with extensive experience in assisting families with death preparation, might think they understood the participants’ stories which could limit deeper exploration during interviews and analysis. Ongoing discussions with NP and QX enhanced researcher reflexivity through challenging assumptions.^26^ A reflexive journal was used to enhance the analysis.^26^

Moreover, the study highlights the importance of cultural context in death preparation, including preparations for the funeral and the deceased’s afterlife. These aspects are often overlooked in clinical practices, which tend to be dominated by Western-centric palliative care knowledge.

A notable strength is the diverse participant pool from seven hospitals across Taiwan, representing various religious beliefs. This diversity enriched the data, facilitating a comprehensive exploration of research questions and improving the transferability of study findings to Taiwan and potentially to Chinese populations, predominantly East Asian societies or Buddhist countries.^26^

Limitations include the study may not fully capture preparedness in contexts involving non-specialist palliative care. The predominance of female participants and adult children of deceased patients, alongside the inclusion of both cancer and non-cancer diagnoses, could impact the study’s transferability.^64^ Further research could explore death preparation among male participants, other family relationships (e.g. spouses) and persons living with chronic conditions before death (e.g. dementia). Including patients’ perspectives in future studies would enhance a more comprehensive understanding of death preparation.

Another potential limitation is the translation of findings from Chinese into English, which may have lost some cultural meaning. However, H-JL and QX spoke both languages, enabling the exploration of language nuances. This was further enhanced by initially coding the data in Chinese and translating it into English later while retaining certain Chinese words.^37^

## Conclusion

Taiwanese family members’ death preparation, influenced by religious beliefs and cultural norms, extends to the time after the death, aiming for a culturally appropriate death. It involves funeral preparations, enhancing the afterlife and maintaining connections. Not showing strong emotions is culturally appropriate and beneficial for the dying relative. These insights inform death preparation in Taiwan and provide perspectives for palliative and end-of-life care beyond Western culture, potentially benefiting Chinese populations, predominantly East Asian societies or Buddhist countries. Future research exploring culturally appropriate strategies for emotional support and maintaining connections before death is needed.

## Supplemental Material

sj-docx-1-pmj-10.1177_02692163241280016 – Supplemental material for ‘A good ending but not the end’: Exploring family preparations surrounding a relative’s death and the Afterlife – A qualitative study Supplemental material, sj-docx-1-pmj-10.1177_02692163241280016 for ‘A good ending but not the end’: Exploring family preparations surrounding a relative’s death and the Afterlife – A qualitative study by Hui-Ju Liang, Qian Xiong, Peng-Chan Lin, Jui-Hung Tsai and Nancy Preston in Palliative Medicine
