# Supplementary material for: ‘A good ending but not the end’: Exploring family preparations surrounding a relative’s death and the Afterlife – A qualitative study
Source: Palliat Med. 2024 Sep 27;38(10):1184–93. doi: 10.1177/02692163241280016 (PMC11613525; doi:10.1177/02692163241280016)

**Supplementary files**

**Supplemental appendices 1:** Participant Information Sheet and Informed Consent Form for Family Caregivers

**Supplemental appendices 2:** Family Caregivers Interview Topic Guide

**Supplemental appendices 3:** Interview Distress Protocol

**Supplemental appendices 4:** Thematic maps

**Supplemental appendices 1: Participant Information Sheet and Informed Consent Form for Family Caregivers**

**Project title**

Preparedness for death and bereavement for family caregivers of terminally ill patients receiving specialist palliative care in Taiwan

**About the researcher**

My name is Huiju Liang. I am conducting this research as a student at Lancaster University, United Kingdom. This research is being conducted as part of my PhD studies.

Email: h.liang3@lancaster.ac.uk, huijuliang.wallis@gmail.com

Mobile number: 0981710900

**NOTE:** Please read the document carefully and ask the researcher any questions about this study if you do not understand any information in this sheet. Thank you for taking your time to read this information sheet.

**About this research**

How families prepare for the death of a loved one is extremely beneficial for family caregivers (e.g., increasing acceptance of a relative’s impending death, ease of transition to bereavement). However, far little attention has been paid to the topic of preparing for the death of a relative for family caregivers, particularly outside of Western countries. To address this issue, this study will invite family members to share their experiences before and after the death of a relative. We will analyse these conversations to have a deeper understanding of such experience and to provide suggestions for future clinical care and research.

**Why have you been approached?**

You have been approached because you were a caregiver of a relative who has passed away within 6-18 months and received palliative care services before his/her death.

**What will happen to you during the study?**

The researcher will ask you to sign an informed consent form after giving you time to consider whether you want to take part in the study. The researcher will arrange a meeting with you for an interview of around 90 minutes, but this could be adjusted according to your need. The interview will take place at a time suitable for you, in a private place such as your home or a restaurant where there is a private place or room. You will be individual interviewed by the researcher with a semi-structured guide only once. During the interview, the researcher will collect general information about you and your relative/significant other you cared for before and then ask you to share about your experience. If we cannot meet in person due to COVID restrictions, the interview may be conducted through virtual meeting software (e.g., Google Meet, or Zoom).

**What is your role/responsibility in this study?**

Participating in this research project is voluntary. A signed copy of the participant information sheet and the informed consent form will be given to you to keep. You have the right to change your mind to freely withdraw from the research project at any stage.

**What are the possible risks of participating in this study?**

You may encounter emotional distress and concerns about discussing the death. If so, the interview can be paused or stopped completely. How much information you share will be your choice and you are free to decline to answer a question. Helpful information about available support and resources will be offered. I would like to contact you in the next day to check if you are OK or need any further support with your permission.

**What are the potential benefits of participating in this study?**

There are no direct benefits of taking part in this study. However, you may have some incidental benefits such as finding the interview therapeutic, having an opportunity for addressing your own grieving issues, having a sense of helping others through contribution to scientific knowledge, and having an enhanced sense of your own worth.

**Cost of participating in this study**

The researcher will not offer financial or non-financial incentive for your participation in this study. However, you may receive a small gift (cookies or similar, less than 200 New Taiwan Dollar).

**Compensation for injury**

If any harm were caused by this research project, except the unavoidable and predicated adverse effects as described earlier, the National Cheng Kung University Hospital will have the responsibility for compensation and provide necessary medical care. Otherwise, no other compensation can be provided. Please do not participate in the study if you do not agree with this.

**Confidentiality of information**

The information you provide will be kept confidential. Your interview recording will be transformed into written notes, removing your identifying information (e.g., name, place) by the researcher and will keep it safe so that others cannot see them except my supervisors. The data including your personal details (signed informed consent, demographic characteristics, audio-recordings, transcriptions) will be secured in a locked drawer or in password-protected folders on a computer; only the researcher can access to them, and they will be safely destroyed after the thesis approval. The non-identifiable information related to the study (e.g., pseudonym transcriptions) will be securely kept on Lancaster University computer system and by the researcher until the year of 2034 and then safely destroyed. During this time, it may be re-analysed to inform new research project that will be approved by the Institutional Review Board. The reporting of the findings of the study will be done to maintain anonymity. Direct quotations from the data you provide will also have been anonymised and be used with your permission. If you disclose any information that makes the researcher thinks that you or someone else are at significant risk, the information will be provided to an appropriate person. If possible, the researcher will tell you before this happens.

**What will happen to the results?**

This study will be completed as part of a PhD thesis that will be publicly available and shared at Lancaster University. Findings may be presented via conferences and peer-reviewed journals articles. In addition, the researcher may share the research findings with the associations related to palliative care education and bereavement to inform future palliative care trainings and clinical practice.

**Voluntary participation**

Participating in this research project is voluntary and is completely up to you to make the decision whether you wish to be involved. You can freely decide whether to take part in this research project. If you change your mind after deciding to participate in the study, you can withdraw from this project at any stage. However, it may not be possible to exclude your data if you wish to withdraw two weeks after the interview.

**Whom to contact in case of any questions**

If you have any questions about this research project or experience adverse effects because of participating in this study, you may contact the researcher Huiju Liang, as detailed above. If you have any questions related to the rights as a participant, you may contact the Institutional Review Board, National Cheng Kung University Hospital. Phone: 06-2353535#3635

Email: em73635@mail.hosp.ncku.edu.tw

Address: No. 138, Shengli Rd, North District, Tainan City, Taiwan 704

**NOTE:** Before you consent to participating in this study, please read the above participant information sheet carefully and mark boxes below if you agree. If you have any questions or queries, please speak to the researcher, Huiju Liang before signing this form.

□ I confirm that I have read the Participant Information Sheet for the above study and fully understand what is expected from me during this research project. I was given the opportunity to ask questions and to have them answered.

□ I consent for the audio recording of my interview, and I understand the researcher will guarantee of the information I provided confidentiality.

□ I understand that my participation is voluntary and that I have the right to withdraw at any time without giving any reasons. I understand that the information I provided for this study will not be excluded two weeks after the interview.

□ I consent to anonymised direct quotations from my interview being used in the thesis, conferences, and publications.

□ I consent to take part in this study, and I conform that I have the signed Participant Information Sheet and Informed Consent Form to keep.

Name and Signature of Participant:

Age of Participant:

Date:

Name and Signature of the researcher:

Date:

**Supplemental appendices 2: Family Caregivers Interview Topic Guide**

- The research question of this interview study:

What are the experiences of preparedness for death and bereavement of family caregivers of terminally ill patients receiving specialist palliative care?

- The interview topics will cover:
- The triggers for conversations about preparing for death and bereavement.
- The components of preparedness for death and bereavement.
- The barriers and facilitators of preparedness for death and bereavement.
- The components of preparedness for death and bereavement benefiting for or worsening bereavement adjustment.

**NOTE:** Question order and wording will be tailored to the circumstances of participants.

1. **Introduction**

- Introduce the researcher self.
- Conversational dialogue to aid developing trust and rapport.
- Completion demographic characteristics related to family caregiver (Appendix 4); fill in by the researcher; demographic characteristics about the deceased will be completed during the interview.

1. **Background**
2. Information about the deceased

- Example: *Please tell me a little about his/her disease.*

1. Information about the family caregiver

- Example: *Please share about your experience of caring for him/her.*

1. **Experience of preparing the death of the relative for the family caregiver**
2. Initial awareness

- Example: *When did/how did you first realised that he/she might be dying（不久人世）or his/her disease status was worse*（病況不好/狀況不好）*?*

1. Tasks completed

- Example: *What tasks did you complete to prepare his/her impending death?*

1. Emotions experienced

- Example: *What were your emotions during this process?*

1. Challenges and resources

- Example: *What challenges did you encounter during this process?*

1. **Professional care offered for helping the family caregiver to prepare the death**

- Example: *What did health care professionals offer in terms of helping you to prepare for his/her impending death?*

1. **Bereavement experience of the family caregiver**

- Example: *Please share about your life during the time of his/her absence.*
- Example: *What effect did the experience of preparing the death have for your bereavement adjustment?*

1. **Conclusion**

- Example: *Is there anything else you would like to say or expand on?*
- Thanks for the participant.

**Supplemental appendices 3: Interview Distress Protocol**

**Step 1: Aware of indications of distress during the interview**

Participants are experiencing a high level of emotional distress or are exhibiting behaviours that show the interview is too stressful such as uncontrolled crying, incoherent speech, and indications of flashbacks.

**Step 2: Immediate response**

- Offer to stop the interview.
- Offer support, allow the participant time to fully express emotions and regroup.
- Assess mental status:

1. Tell me what thoughts you are having.
2. Tell me what you are feeling right now.
3. Do you feel you are able to go on about your day?

- Determine if the participant is experiencing acute emotional distress beyond what would be normal expected in an interview about bereavement experience.

**Step 3: Taking actions based on situation of the participant**

- Continue the interview if the participant feels that he/she can carry on.
- Stop the interview completely if the participant feels that he/she is unable to continue. The researcher accompanies him or her until he/she feels okay and contact his/her family member or mental health provider if needed.

**Step 4: Follow up**

- Offer helpful information about available support and resources if needed.
- Encourage the participant to seek professional help (e.g., mental health provider).
- The researcher will contact the participant the next day to see if he/she is okay, with his/her permission.

**Supplemental appendices 4: Thematic maps**


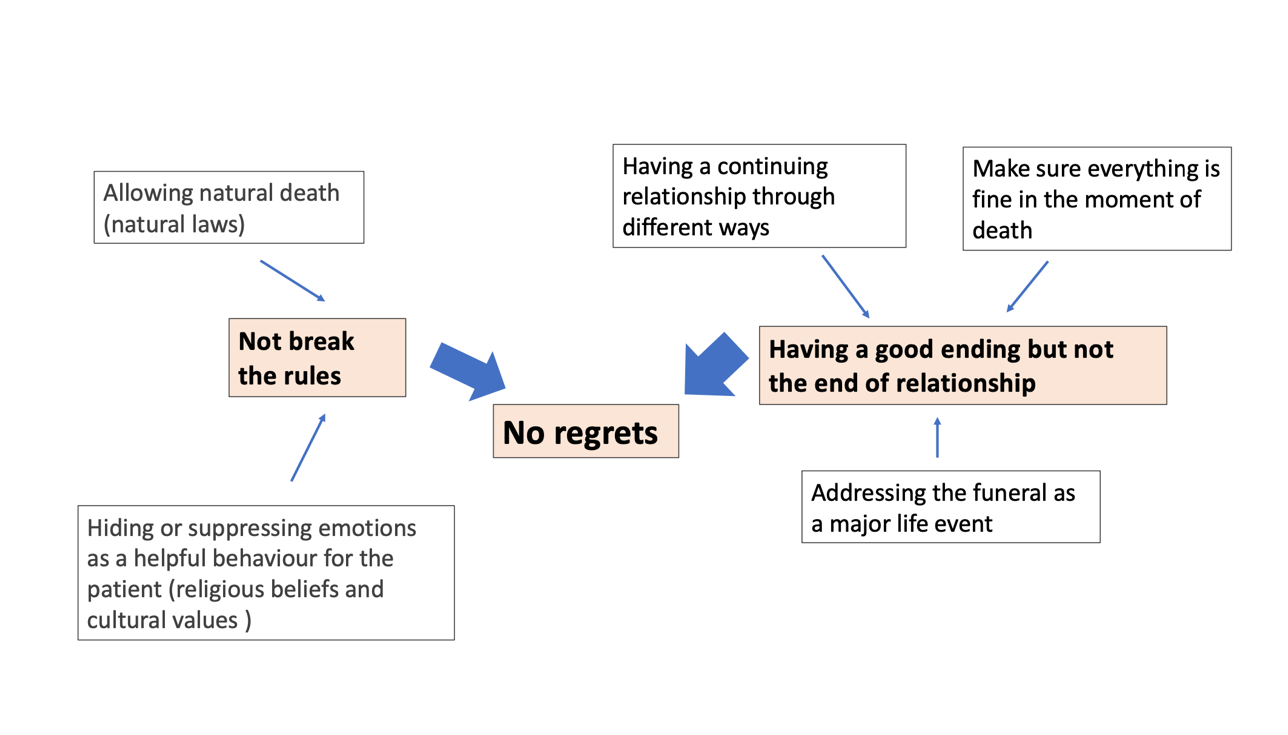


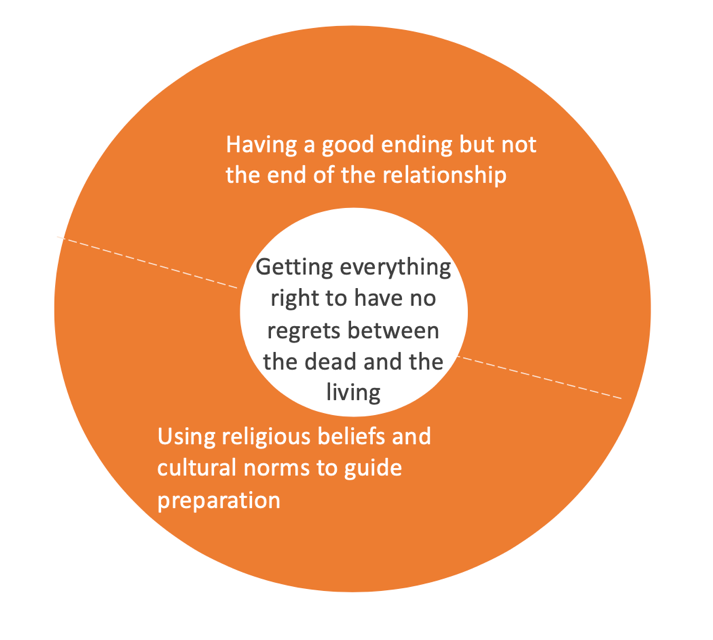

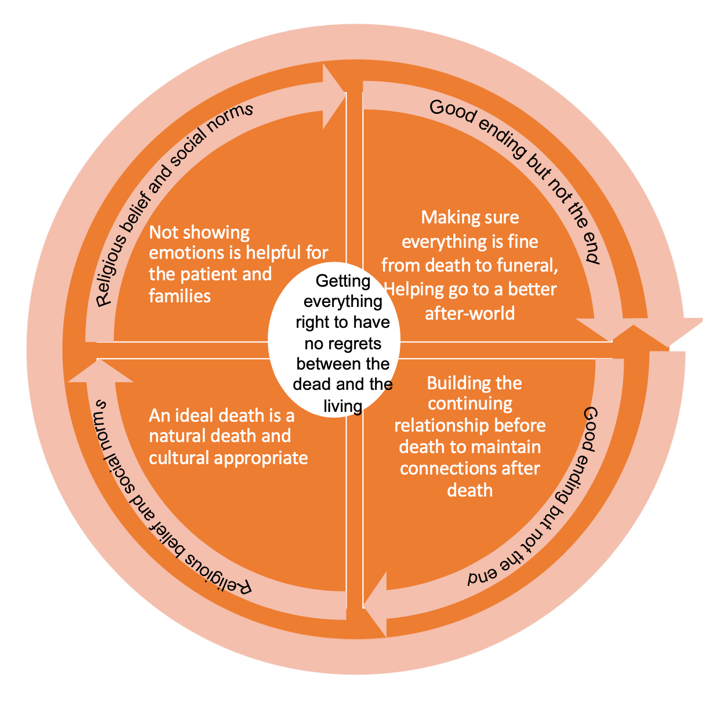

Supplement: sj-docx-1-pmj-10.1177_02692163241280016 – Supplemental material for ‘A good ending but not the end’: Exploring family preparations surrounding a relative’s death and the Afterlife – A qualitative study [file sj-docx-1-pmj-10.1177_02692163241280016.docx]
